# Supplementary figures and images for: Crimean-Congo hemorrhagic fever virus NSm protein inhibits the type I interferon signaling by binding to STAT2
Source: PLoS Negl Trop Dis. 2025 Nov 4;19(11):e0013695. doi: 10.1371/journal.pntd.0013695 (PMC12594364; doi:10.1371/journal.pntd.0013695)

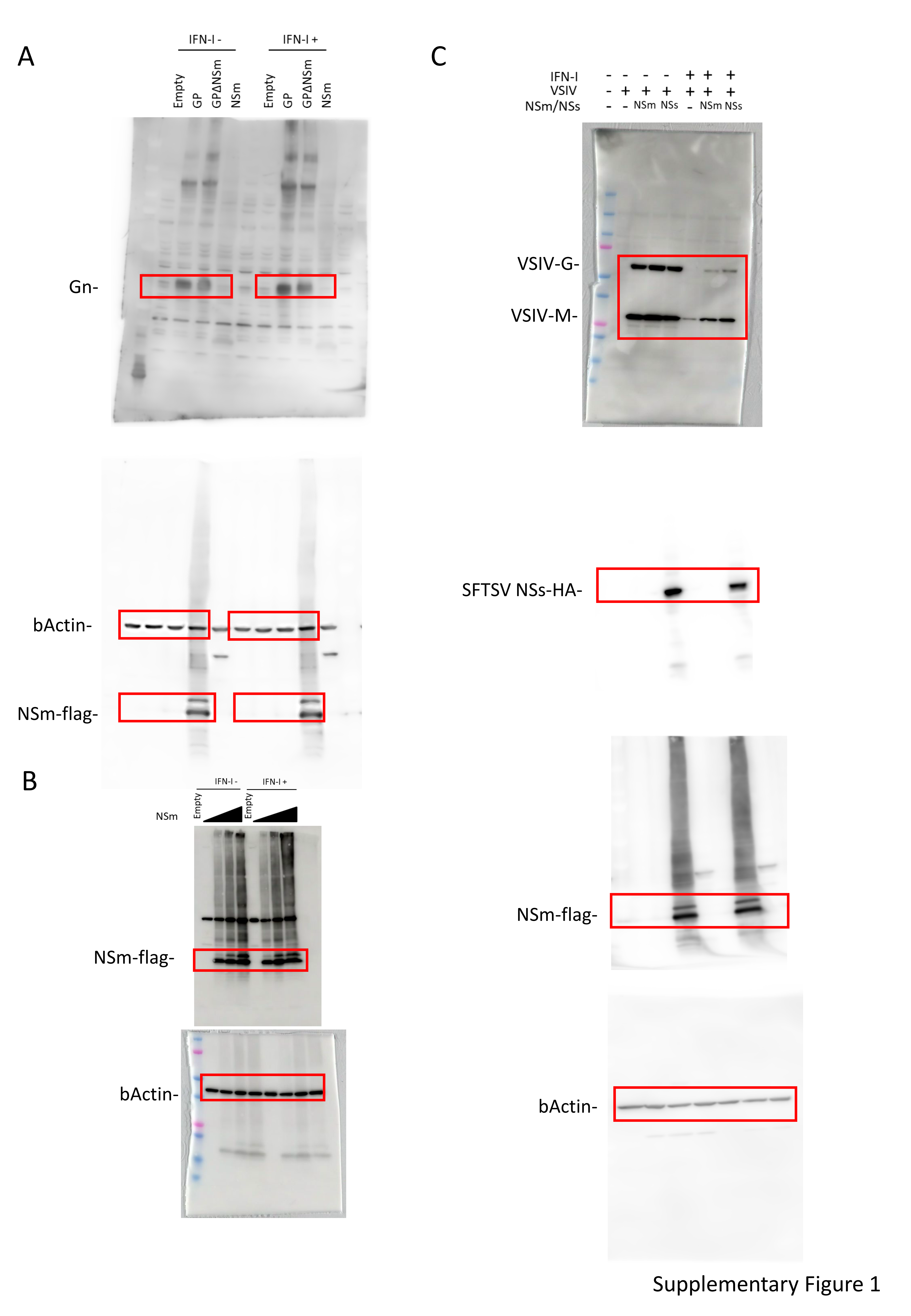

Supplement: S1 Fig — (B) Original (uncropped) blots of Fig 1B. (C) Original (uncropped) blots of Fig 1C. (D) Original (uncropped) blots of Fig 1D. Cropped regions are indicated by red squares. (TIF) [file pntd.0013695.s001.tif]

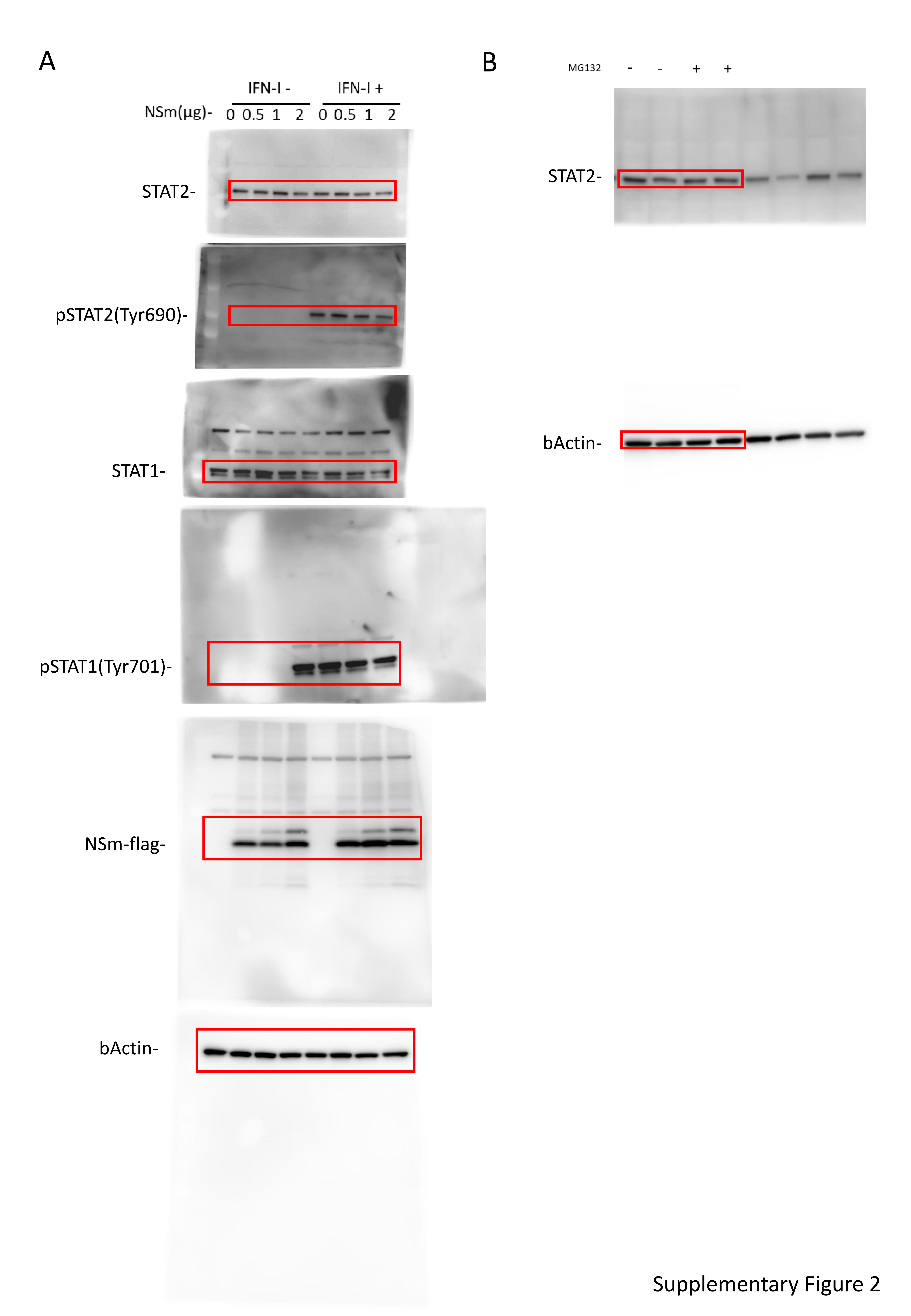

Supplement: S2 Fig — (B) Original (uncropped) blots of Fig 2C. Cropped regions are indicated by red squares. (TIF) [file pntd.0013695.s002.tif]

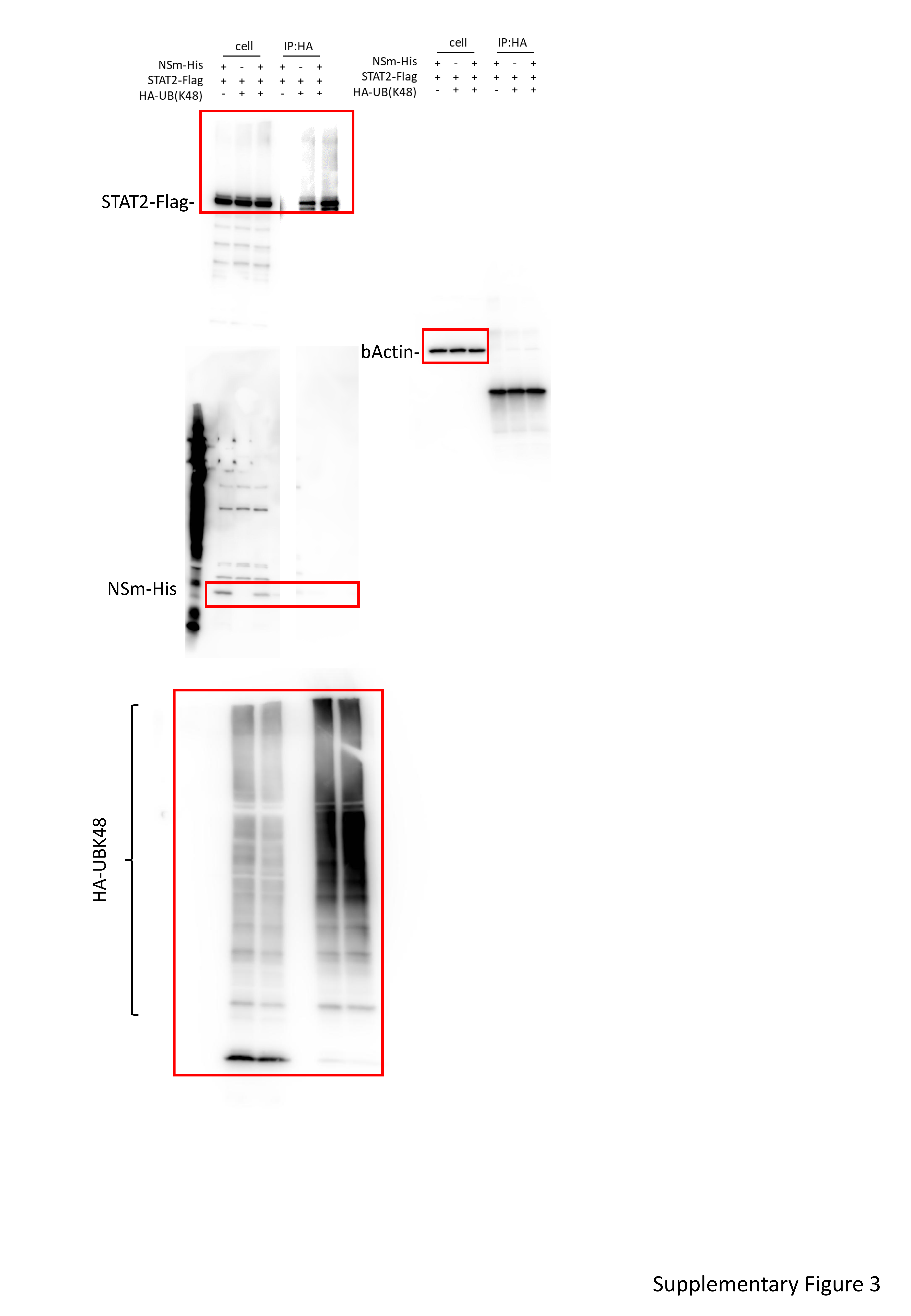

Supplement: S3 Fig — Cropped regions are indicated by red squares. (TIF) [file pntd.0013695.s003.tif]

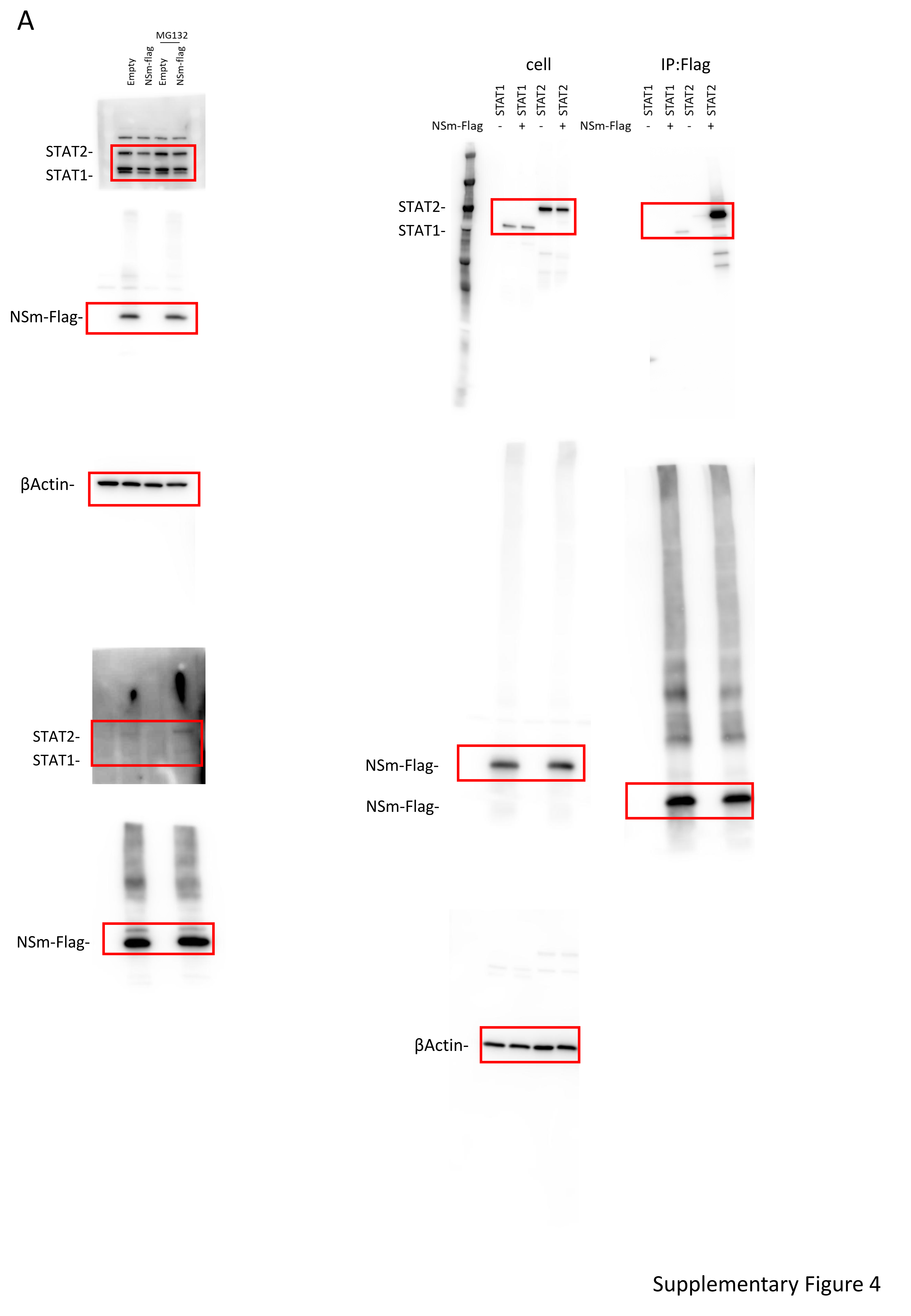

Supplement: S4 Fig — (B) Original (uncropped) blots of Fig 3B. Cropped regions are indicated by red squares. (TIF) [file pntd.0013695.s004.tif]

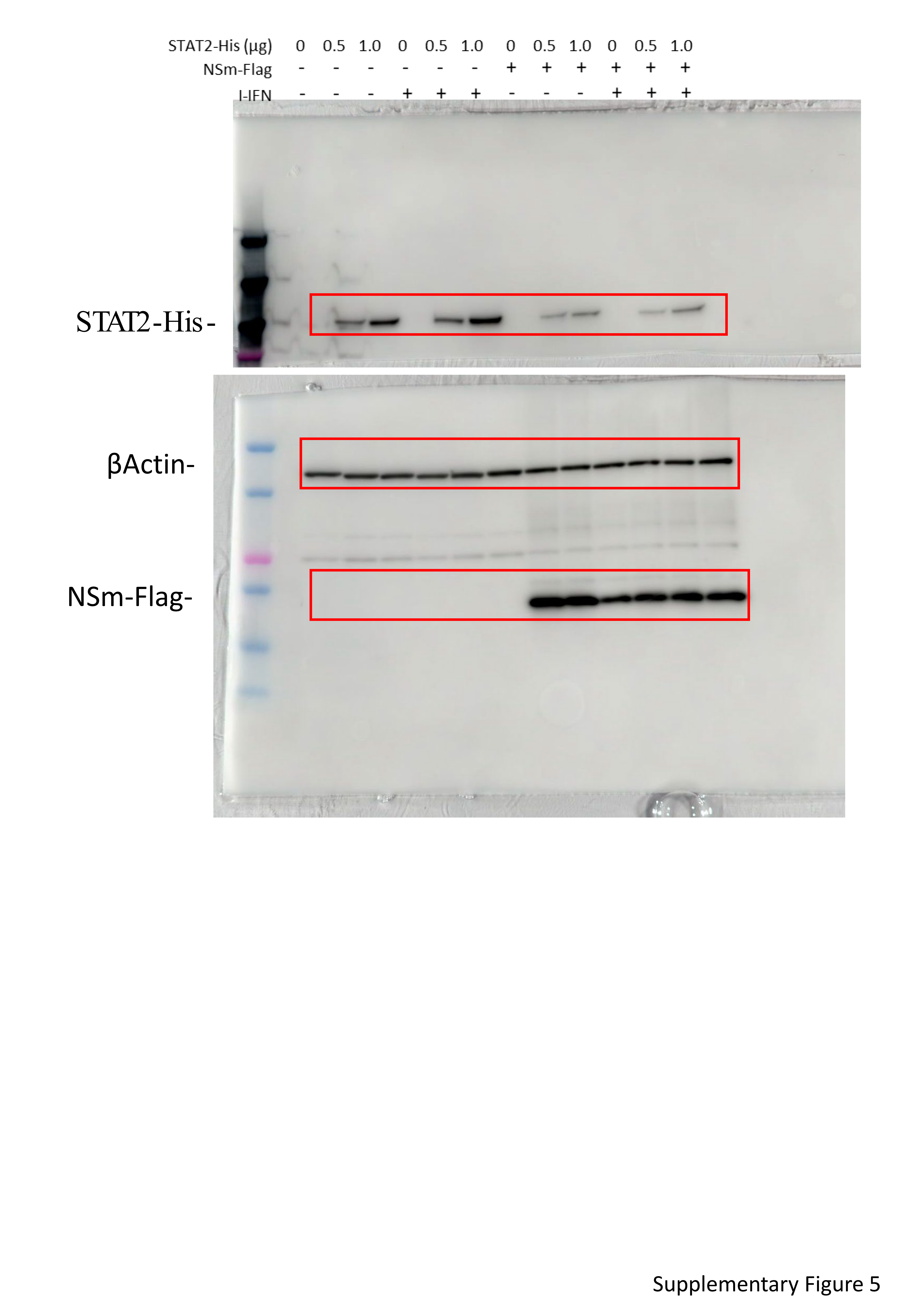

Supplement: S5 Fig — Cropped regions are indicated by red squares. (TIF) [file pntd.0013695.s005.tif]

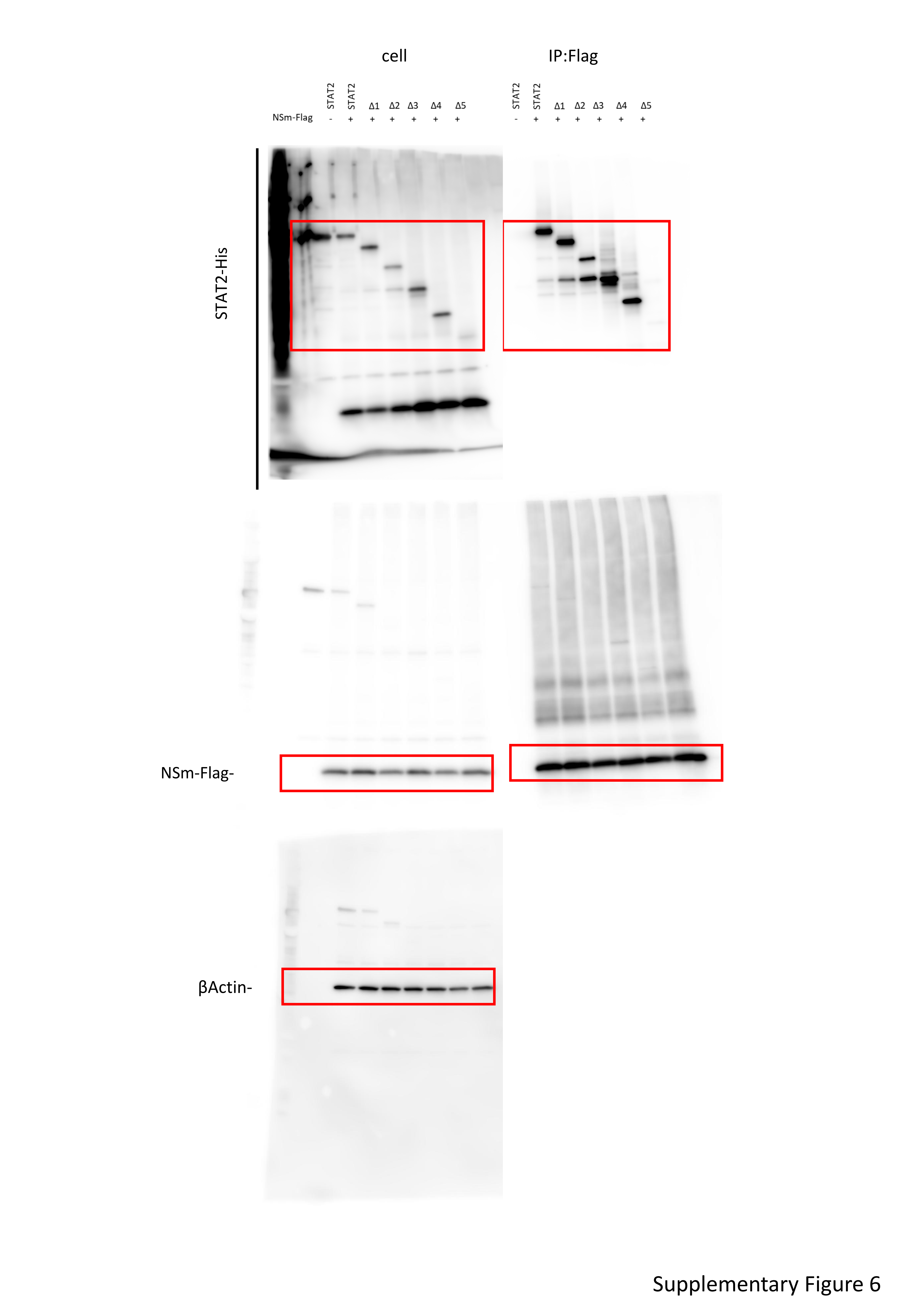

Supplement: S6 Fig — Cropped regions are indicated by red squares. (TIF) [file pntd.0013695.s006.tif]

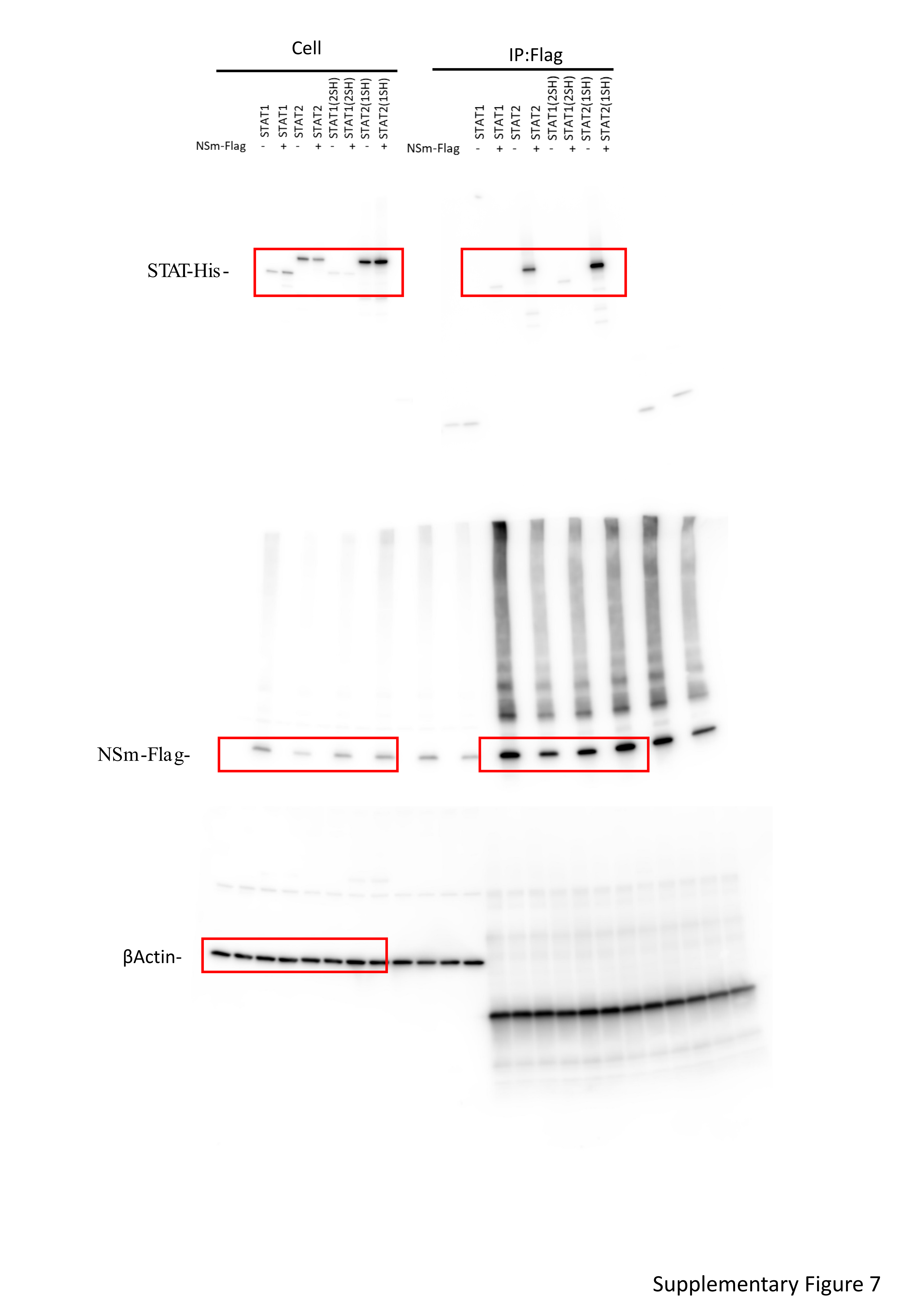

Supplement: S7 Fig — Cropped regions are indicated by red squares. (TIF) [file pntd.0013695.s007.tif]
