# Supplementary material for: Crimean-Congo hemorrhagic fever virus NSm protein inhibits the type I interferon signaling by binding to STAT2
Source: PLoS Negl Trop Dis. 2025 Nov 4;19(11):e0013695. doi: 10.1371/journal.pntd.0013695 (PMC12594364; doi:10.1371/journal.pntd.0013695)
Supplement: Attachment — Submitted filename: Response to Editor and Reviewers RS final.docx [file pntd.0013695.s009.docx]

**Response to Editor**

Thank you very much for your comments regarding our manuscript. We revised our manuscript according to your comments.

Comments:
1) Explore opportunities to validate STAT2 degradation or NSm-STAT2 interactions in the context of authentic CCHFV infection (e.g., using WT vs ΔNSm virus), or in a reverse genetics system if possible.

Re: Thank you for your advice. We understand that it is important to make sure the results using infectious virus. However, we cannot use replication-competent CCHFV at present, since our BSL4 facility in Nagasaki University has not started BSL-4 operation.

2) Consider testing species-specific STAT2 interactions to support the hypothesis that NSm contributes to host range restriction.

Re: Thank you for your suggestion. We will investigate the relationship between the species-specificity of CCHFV and NSm function using replication-competent CCHFV and a mouse model such as human STAT2 knock-in mice as a next study. Therefore, in this paper, we have only demonstrated the relationship between NSm and human STAT2.

3) Strengthen the evidence for STAT2 re-localization using quantitative co-localization analysis and higher-resolution microscopy.

Re: We apologize for the confusion. All images were acquired using a confocal microscope. Therefore, we described an additional information in Materials and methods (page 11, line 167). In the revised version, we increased the resolution of images. And we added the distribution image of each signal (Figs. 3 and 5). To investigate the intracellular co-localization of proteins, the same method was used in our previous studies (Yoshikawa *et al* 2019; Yoshikawa *et al* 2023).

4) If proposing a role for NSm in E3 ligase recruitment, include supporting bioinformatic analysis (e.g., motif scans), or clearly frame this as a hypothesis.

Re: According to your suggestion, we searched the motifs related to protein degradation using Eukaryotic Linear Motif (ELM) resource (<http://elm.eu.org/>). And we discussed the results based on the findings (Pages 20-21, lines 335-345).

5) A figure showing NSm sequence conservation across diverse CCHFV strains would help contextualise its functional relevance.

Re: According to your suggestion, we added the figure showing the NSm sequence alignment of various CCHFV strains (Fig. 7)

**Response to Reviewer #1**

Thank you very much for your comments regarding our manuscript. We revised our manuscript according to your comments.

1) There are multiple issues regarding the presented data.
Figure 1D: Although the effect of SFTSV NSs (used as a positive control) is clear, the inhibitory activity of NSm is not convincing. It could be due to low level of NSm expression. I encourage the authors to repeat the experiment and show quantitative data.

Re: As you pointed out, in the original version, the expression level of NSm was low. According to your suggestion, we repeated this experiment and quantified the inhibition rate of VSIV G and M protein levels by INF-I. In the revised version, we concluded that NSm recovered VSIV G and M protein expression to the same extent as SFTSV NSs (Page 13, lines 205-208; Fig. 1D).

2) Figure 2A and lines 207-8 “the STAT2 protein expression level decreased in an NSm concentration–dependent manner”: It is not convincing with the presented image. The band intensity of STAT2 is very weak compared to STAT1 unlike the image shown in Fig 3A. The data should be repeated and quantitated. The same for phosphorylated STAT2.

Re: According to your suggestion, we repeated the immunoblotting and quantified the expression levels of each protein (Fig 2A).

3) Figure 2B and lines 211-4” The quantitative real time RT-PCR results indicated that STAT2 mRNA levels were comparable in the presence and absence of NSm in HEK293T cells (Fig. 2B). These results suggest that NSm downregulates STAT2 at the protein but not mRNA level”: It is unclear how much cDNAs were transfected in this experiment. The authors need to use the same dose of cDNAs to compare their impact on protein and mRNA expression.

Re: In the revised version, we described the amount of the NSm expression plasmid transfected. The amount of the plasmid transfected was the same in the experiments shown in Fig. 2A and Fig. 2B (page 14, lines 220-222; page 31, line 513. Fig. 2A).

4) Figure 2D: There are a couple of problems in this figure. 1) In the cell lysate (left), there is no difference in STAT2-Flag expression between NSm-His expressing and not expressing cells. 2) In IP material, why more STAT2-Flag (UB unconjugated) was detected in NSm-His expressing cells than non-expressing cells? Was this experiment conducted in the presence of MG132? The authors should consider performing IP using anti-Flag Ab to collect STAT2-Flag and detect UB(K48) by anti-HA Ab.

Re: We apologize for the confusion. To efficiently detect ubiquitinated STAT2, we performed this experiment in the presence of MG132. In the revised version, we added its information (page 15, line 236). We demonstrated the co-IP assay using anti-Flag antibodies. However, we chose the co-IP assay using anti-HA antibodies in this study, since the co-IP assay using anti-Flag antibodies did not work well.

5) Figure 3C and line 240. ”NSm was co-localized with STAT1 and STAT2”. : The IF image using conventional microscope at this magnification does not provide convincing data for protein co-localization. Analysis using confocal microscope is recommended.

Re: We apologize for the confusion. All images were acquired using a confocal microscope. Therefore, we described an additional information in Materials and methods (page 11, line 167). In the revised version, we increased the resolution of images. And we added the distribution image of each signal (Fig. 3). To investigate the intracellular co-localization of proteins, the same method was used in our previous studies (Yoshikawa *et al* 2019; Yoshikawa *et al* 2023).

6) Figure 4: Western blot data showing the overexpression of STAT2 should be included in this figure.

Re: According to your suggestion, we added the Western blot data showing the overexpression of STAT2 (Fig. 4 (lower panel)).

7) Figure 5: As explained above, the presented images at this resolution are not convincing to make conclusion stated in lines 250-256.

Re: As stated in 5), we revised the data and added the distribution image of each signal (Fig. 5).

8) Figure 6B: Are these experiments conducted in the presence of MG132? The STAT2 level in the lysate seems to be the same regardless the presence of NSm-Flag.

Re: We apologize for the confusion. We performed the immunoblotting again. In the revised version, the expression level of STAT2 in the presence of NSm was lower than that in the absence of NSm (Fig. 6B).

9) Figure 6F: the data seem to disagree with the image shown in Figure 6D.

Re: We adjusted the brightness of the image (Fig. 6D). And, we recalculated the protein expression rate in the presence of NSm, but obtained the same result. In the revised version, the raw data and original blot were shown in S1 Data or Supplementary Figure 7, respectively.

**Response to Reviewer #2**

Thank you very much for your comments regarding our manuscript. We revised our manuscript according to your comments.

1) manuscript concludes that NSm interacts with STAT2; however, the co-immunoprecipitation (co-IP) data presented in Figure 3A show only a modest level of STAT2 pull-down, with no clear enrichment compared to the input lysate. While Figure 3B demonstrates a stronger interaction, this result is based on overexpression of STAT2, which may not reflect physiological conditions. The authors should address this discrepancy and discuss the limitations of relying on overexpression systems, particularly in interpreting the strength and specificity of the NSm–STAT2 interaction.

Re: In the revised version, we added the following sentences in the Discussion. “In this study, we demonstrated that NSm bound to STAT2 (Figs. 3A and B). However, the amount of endogenous STAT2 bound to NSm was much less than that of exogenous STAT2. This may be explained by the differences in the protein expression level between exogenous STAT2 and endogenous STAT2. Although the protein overexpression experiments using expression plasmids do not necessarily mimic the endogenous protein expression levels in the cell, both the expression level of endogenous and exogenous STAT2 was reduced by NSm (Figs. 3A and B). These results indicate that NSm specifically interacts with STAT2. Animal experiments using mice, such as human STAT2 knock-in mice, would be required to investigate whether STAT2 degradation activity by NSm is involved in CCHFV pathogenicity and fully understand the relationship between STAT2 and NSm *in vivo*.” (page 20, lines 320-330)

2) The author used various protocol for IFN treatment, those variations are not stated in the text and make the figure/result part difficult to interpret for the reader. I.e Figure 1A is a WB performed at 18h post-IFN treatment, when Figure 2A is 30 min after IFN treatment.

Re: According to your suggestion, we described the protocol for IFN treatment in the main text (page 12, lines 189-190; page 14, lines 217-219; page 15, lines 257-259).

3) Could the author explained the rationale behind the use of HEK293T cells (which lack endogenous IFN production and are not the natural targets of CCHFV.), why they did not choose IFN competent cells like A549? or type I IFN deficient cells like Vero E6 to make a comparison?

Re: HEK293T cells are IFN-I-competent cells and susceptible to CCHFV infection [Yoshikawa R *et al*., 2023; Földes K *et al*., 2020]. We added this information in main text (page 12, lines 188-189). The transfection efficiency into HEK293T cells is higher than that into cells such as A549. Therefore, HEK293T cells are commonly used to analyze the relationship between viral proteins and IFN-I signaling (Ning YJ *et al*., 2015; Chaudhary V *et al*., 2015; Rezelj VV *et al*., 2017; Kitagawa Y *et al*., 2019; Feng K *et al*., 2019; Ren W *et al*., 2024; Miorin L *et al*., 2019).

3) Could the author explained the low level of NSm expression in HEK293T cells? Could we have an additional figures showing level of expression on >100 cells?

Re: To show both NSm-expressing cells and NSm-non-expressing cells in the same field, we controlled the expression level of NSm by adjusting the amount of plasmid used for transfection. When many cells are stained, it may be impossible to distinguish between specific and non-specific staining. In addition, to investigate the intracellular co-localization of proteins, the same method was used in our previous studies (Yoshikawa *et al* 2019; Yoshikawa *et al* 2023).

4) Discrepancies between many blot concerning NSm-FLAG expression, i.e Fig 1A and 1B IFN- and IFN + show very different band intensity in those 2 blots? No mention of this in the results part or in the discussion.

Re: We performed the immunoblotting again. In Fig.1B, to clearly show the band of NSm in 0.5ug of the NSm plasmid, the membrane was exposed to the substrate longer than Fig. 1A.

5) Supplementary data of full blots should be provided for all Western Blot presented here as some discrepancies are observed (including in the same size band e.g; Figure2A NSm-FLAG empty is white when background of other band is grey)

Re: According to your suggestion, in the revised version, we added the data of full blots as supplementary figures.

Minor comments

6) Figure 1D: Could author explained the low level of NSm-FLAG? (When compare to Figure 1A this looks strange).

Re: We performed this experiment again. In the revised version, we confirmed that the expression levels of NSm in Fig. 1A and Fig. 1D were comparable.

7) Figure 2B: could the increase of mRNA level at 48h be explained in the text?

Re: In the revised version, we added the following sentences in the Discussion.” Interestingly, we demonstrated that NSm increased mRNA expression level of STAT2 (Fig. 2B). NSm may have a function that regulates RNA expression in the host, although we cannot explain this result at present.” (page 20, lines 331-333)

8) Figure 2D: make the labelling of the panel clearer is the lower part HA mediated Immunoprecipitation?

Re: We apologize for the confusion. The left panel indicated the results of whole cells and the right panel indicated the results of co-IP. In the revised version, we modified the Fig. 2D.

9) Figure 6B: Could the author address the low level of expression of STAT2 DELTA5? And consequence for the pull down?

Re: In the revised version, we added the following sentences in Results section. “As shown in Fig. 6B, the expression level of Δ5 was lower than that of the others. The cause of this is thought to be the instability of protein due to deletion. In this condition, NSm bound to STAT2Δ1, Δ2, Δ3, and Δ4, but not Δ5 (Fig. 6B). However, it was still unclear whether the binding of NSm and STAT2 was mediated by SH domain, since the expression level of Δ5 was extremely low.” (page 17, lines 276-281)

10) Line 116: what is LT-1 cells?

Re: We corrected it. (page 7, lane 116)

11) Line 145: What is IFN A/D?

Re: We added the information on IFN-αA/D. (page 9, line 144)

12) Line 194: could you add a reference regarding the type I IFN treatment

Re: We added the reference. (page 13, line 200)

13) Line 202: Could you address the low level of NSm-FLAG expression in cells?

Re: Please see our reply to the comment 6).

14) Could you add a reference for the protein degradation statement?

Re: We added the reference. (page 14, line 232)

15) Line 222-224: Could the author described the results observed, was hard as non-specialist to read that panel without guidance

Re: In the revised version, we added the background information about this experiment to confirm K48-linked ubiquitination and details of the results. (pages 14-15, lines 230-234; page 15, lines 236-238)
