# Supplementary material for: Crimean-Congo hemorrhagic fever virus NSm protein inhibits the type I interferon signaling by binding to STAT2
Source: PLoS Negl Trop Dis. 2025 Nov 4;19(11):e0013695. doi: 10.1371/journal.pntd.0013695 (PMC12594364; doi:10.1371/journal.pntd.0013695)
Supplement: Attachment — Submitted filename: Response to Reviewers.docx [file pntd.0013695.s010.docx]

**Response to Reviewer #1**

Thank you very much for your comments regarding our manuscript. We revised our manuscript according to your comments.

1) The authors may comment about the upper band of NSm-Flag detected in Figure 1A, B, D, and Fig 2A.

Re: We think that this upper band most likely represents a post-translationally modified form of the NSm protein, such as glycosylation or phosphorylation. We added a brief explanation in the Results section (page 13, lines 205-208).

**Response to Reviewer #2**

Thank you very much for your comments regarding our manuscript. We revised our manuscript according to your comments.

1) I understand your rationale for controlling NSm expression levels by adjusting plasmid amounts. However, I believe including both mock-transfected and NSm-transfected cells would be a better strategy to demonstrate the efficiency of your transfection protocol in HEK293T cells. Could you please ensure that you add the specific amount of NSm plasmid used for transfection in your figure legend?

Re: According to your comment, we described the amount of the NSm plasmid used for transfection in the figure legend (page 34, lines 555-556; page 35, lines 576-577).

Minor Comments:
2) Line 131: Please provide the complete reference for LT-1, especially since you mention following the manufacturer's protocol. Who is the manufacturer?

Re: We added the catalog number in Materials and methods section, and corrected the product name and the company name (page 8, lines 120-121).

3) Line 178: Please state IFA in full at first mention.

Re: Thank you for your comment. We have already stated "IFA" in full as immunofluorescence assay at its first mention in the manuscript (page 8, line 125).

4) Lines 214-215: Could you please clarify how you assess ISRE gene activation in HEK293T cells? Which genes did you use, and what were the upregulation levels? This is unclear from both the text and the figure. I think providing more details about your dual-luciferase assay would help the reader. Additionally, in your figure axis labels, please make it clear that you are measuring luciferase activity.

Re: Thank you for your valuable comments. We assessed ISRE activation in HEK293T cells using a dual-luciferase reporter assay. Specifically, the cells were transfected with a plasmid containing the ISRE promoter upstream of the firefly luciferase gene, along with a Renilla luciferase plasmid as an internal control. The relative luciferase activity was calculated by normalizing firefly luciferase signals to Renilla luciferase signals.

We did not measure endogenous ISRE-regulated gene expression in this assay; rather, the luciferase activity served as a proxy for ISRE promoter activation. We clarified this methodology in the Materials and methods section (page 7, lines 107-113).

Additionally, we have revised the axis labels in the figures to clearly indicate that the values represent relative luciferase activity (Figures 1A, 1B, and 4).

5) Line 222: Please state the acronym VSIV in full at first mention.

Re: Thank you for your comment. We have already stated "VSIV" in full as vesicular stomatitis Indiana virus at its first mention in the manuscript (page 7, line 102).

6) Figure 1D: You mention that HEK293T are IFN-competent cells, but VSIV infection, which is sensitive to IFN action, is inhibited only after addition of IFN. Is this because you are examining early time points?

Re: Thank you for your insightful observation. Yes, the inhibition of VSIV infection was observed only in HEK293T treated with IFN, likely because the time points examined in our assay were relatively early (6 hpi) and did not allow sufficient time for endogenous IFN production and signaling to reach levels that could effectively suppress viral replication.

7) Line 299: Please state what TGN stands for at first mention.

Re: Thank you for your comment. We have already stated "TGN" in full as trans-Golgi network at its first mention in the manuscript (page 8, line 130).

8) Line 303: Do you have a quantitative measure for NSm localization in the TGN? The use of the word "partially" is too vague.

Re: Thank you for your comment. Although we did not perform a quantitative co-localization analysis in this study, we provided representative images showing the spatial distribution of each signal. Based on these images, we observed that a portion of the STAT2 signal overlapped with the TGN marker signal only in the presence of NSm. Therefore, we used the term "partially localized" to describe this qualitative observation. We have clarified this point in the revised manuscript to improve clarity (pages 17-18, lines 284-288).

9) Line 374: Please add a reference to support the following statement: "NSm may have a function that regulates RNA expression in the host."

Re: Thank you for your comment. The statement regarding NSm’s potential role in regulating host RNA expression is based on our interpretation of the observed experimental results and is currently speculative. To avoid confusion, we have revised the wording to clarify that this is a hypothesis derived from our findings, and we have removed the implication that it is supported by existing literature (page 21, lines 349-353).

10) All good. Addition of manufacturer reference for all chemical and reagents used in the study will help for reproducibility.

Re: Thank you for your suggestion. We have revised the manuscript to include the manufacturer information for all chemicals and reagents used in the study. These updates have been incorporated into the Materials and methods section.
